# Succinic semialdehyde dehydrogenase deficiency: exploring the relationship between *ALDH5A1* variants and molecular effect on SSADH

**DOI:** 10.1186/s13023-026-04409-z

**Published:** 2026-05-30

**Authors:** Dandan Yan, Xiangyu Liu, Chunyu Gu, Yingzi Cai, Wenxuan Fan, Chunhua Zhang, Dong Li, Jianbo Shu, Hanjie Wang, Chunquan Cai

**Affiliations:** 1 Children’s Hospital, Tianjin University (Tianjin Children’s Hospital), Tianjin, 300134 China; 2 Tianjin Key Laboratory of Birth Defects for Prevention and Treatment, Tianjin, 300134 China; 3 Tianjin Pediatric Research Institute, Tianjin, 300134 China; 4 Graduate College of Tianjin Medical University, Tianjin, 300070 China; 5 Graduate College of Tianjin University, Tianjin, 300072 China; 6 Department of Neurology, Children’s Hospital, Tianjin University (Tianjin Children’s Hospital), Tianjin, 300134 China; 7 Department of Research and Development, MILS International, Kanazawa, Ishikawa 912-8105 Japan; 8 School of Life Sciences, Tianjin University and Tianjin Engineering Center of Micro-Nano Biomaterials and Detection-Treatment Technology, Tianjin Key Laboratory of Function and Application of Biological Macromolecular Structures, Tianjin, 300072 China

**Keywords:** SSADHD, *ALDH5A1*, Protein structure and function, ACMG/AMP

## Abstract

**Background:**

Succinic semialdehyde dehydrogenase deficiency (SSADHD), caused by variants in *ALDH5A1*, is a rare inherited neurometabolic disorder with phenotypic heterogeneity. To clarify the pathogenicity of *ALDH5A1* variants, we systematically investigated their effects on succinic semialdehyde dehydrogenase (SSADH) structure and function.

**Methods:**

We obtained the clinical and molecular characteristics of 12 probands. We further augmented the pathogenicity dataset for 14 variants using multiple in silico tools under the American College of Medical Genetics and Genomics/Association for Molecular Pathology (ACMG/AMP) guideline. More importantly, we provided quantitative data on protein expression and residual enzyme activity for SSADH, enabling robust assignment of ACMG PS3/BS3 codes. Finally, we integrated 3D structural modelling with changes in physicochemical properties to butter elucidate how individual variants impair SSADH function.

**Results:**

Patients with SSADHD exhibit varying degrees of epilepsy, intellectual disorders, developmental delay, and hypotonia. Among the 14 variants, 9 affect SSADH expression level of protein, and 9 affect SSADH enzyme activity. After acquiring functional experiments’ results, PS3 was assigned to 10 variants and BS3 to 1; more notably, 8 variants obtained more plentiful pathogenic evidence. Intriguingly, we also investigated the underlying mechanisms using protein-based prediction techniques, 2 of 14 variants influenced binding of SSADH to small molecules, whereas 3 of 14 variants led to instability. Besides, as the SSADH with variants in the oligomerization domain accumulates in the tetramer, the tetramer becomes increasingly unstable.

**Conclusion:**

In this study, we illustrated that 14 *ALDH5A1* variants affected the molecular structure and function by diverse mechanisms, and with varying degrees, which could help us to gain a deeper understanding of these variants by providing reasonable evidence based on the PS3/BS3 codes in the ACMG guidelines. Essentially, this pathogenic classification facilitates the diagnosis of SSADHD in future genetic testing processes.

**Supplementary Information:**

The online version contains supplementary material available at 10.1186/s13023-026-04409-z.

## Introduction

Since first described in 1981 [1], SSADHD has been reported in hundreds of patients and *ALDH5A1* pathogenic variants over 450 countries worldwide [2]. Studies have shown that pathogenic variants are found throughout all exons, with missense variants representing the majority, whereas deletions, insertions, and splice site variants constitute a minor fraction [3]. Succinic semialdehyde dehydrogenase (SSADH) is encoded by *ALDH5A1*, located in the 6p22.3 region; the complete open reading frame is 1605 bp and encodes 535 amino acids. *ALDH5A1* comprises 10 exons and spans over 38 kb; its variants may cause SSADHD [4]. SSADH is a mitochondrial protein with four domains: mitochondrial targeting signal domain, NAD^+^-binding N-terminal domain, catalytic domain, and oligomerization β-structured domain. SSADH catalyzes the oxidation of succinic semialdehyde (SSA) to succinic acid in leukocytes [5, 6]. The loss or variation of several well-conserved glycine residues, previously reported as critical for enzyme function, led to nearly complete ablation of enzyme activity [7].

**Fig. 1:**
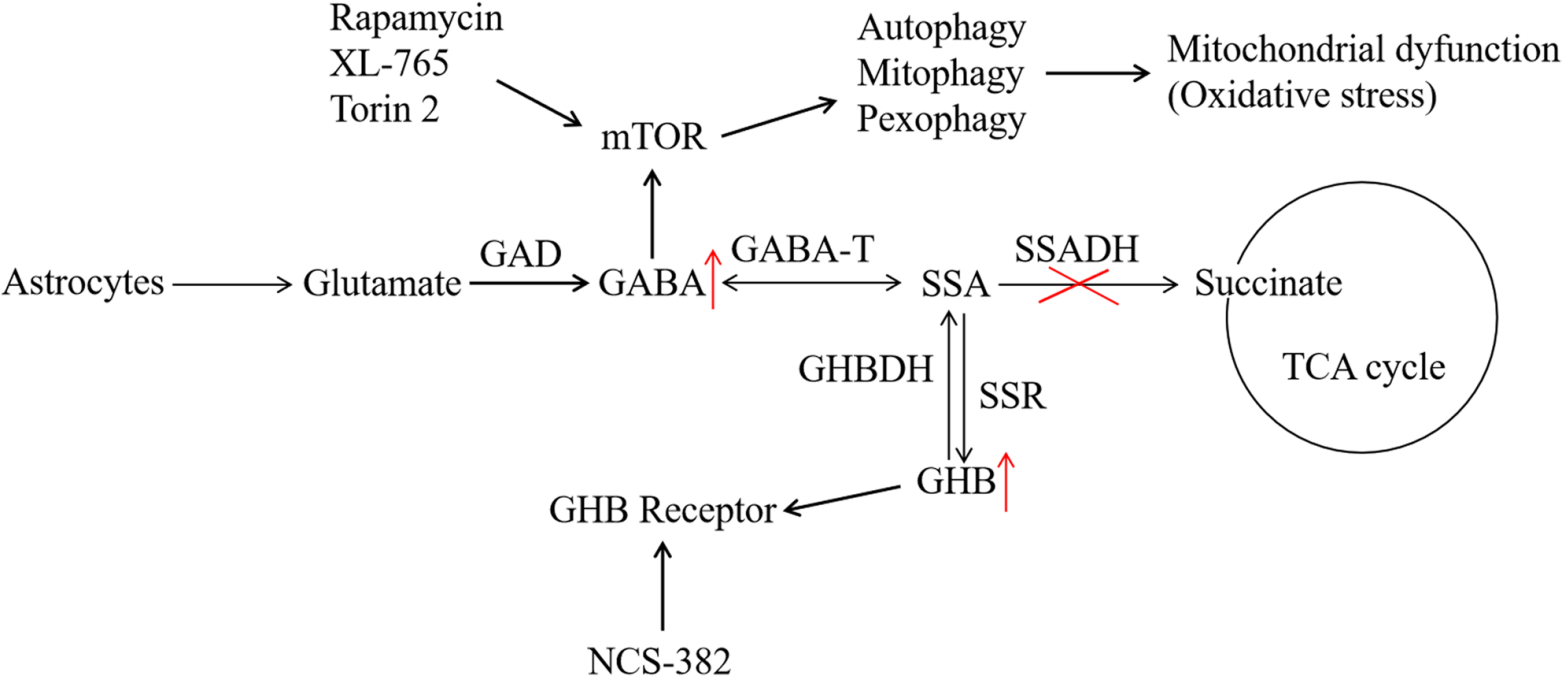
Flowchart of GABA metabolism and SSADH deficiency. GAD, glutamic acid decarboxylase; GABA, aminobutyrate; GABA-T, GABA-transaminase; SSR, succinic semialdehyde reductase; GHBDH, hydroxybutyrate dehydrogenase; GHB gamma-hydroxybutirate; SSADH, succinic semialdehyde dehydrogenase. XL-765 and Torin 2 represent potent inhibitors of mTOR. NCS-382 represents a high-affinity ligand of receptor(s) binding GHB and a possible substrate inhibitor for GHB transport. When SSADH deficiency (indicated by the crossed block), the bypass products GABA and GHB increase (indicated by the upward arrows)

SSADHD—also known as 4-hydroxybutyric aciduria or γ-hydroxybutyric aciduria—is a rare autosomal-recessive disorder marked by markedly elevated levels of γ-aminobutyric acid (GABA) and its metabolites [8]. GABA, an inhibitory neurotransmitter, is synthesized from the excitatory neurotransmitter glutamate by the action of glutamic acid decarboxylase. In the canonical pathway, GABA is transaminated to SSA by GABA-transaminase, and SSA is subsequently oxidized to succinate by SSADH for entry into the Krebs cycle [9]. SSADH deficiency disrupts the GABA degradation pathway (Fig. 1); SSA is reduced to γ-hydroxybutyrate (GHB) by aldo-keto reductase, resulting in accumulation of GABA and GHB in patients’ physiological fluids. GHB, a well-known GABA analogue with neuromodulatory activity, causes mild-to-severe neurological deficits at elevated concentrations [10].

The phenotypic spectrum of SSADHD is extensive, ranging from mild intellectual disability to severe neurological defects, including developmental delay and early-onset hypotonia, later-onset expressive language impairment and obsessive-compulsive disorder, hyporeflexia, non-progressive ataxia, and epilepsy [8]. The majority of patients suffer hypotonia and developmental delay, with approximately 50% having epilepsy [11]. Before SSADHD was diagnosed, some patients died suddenly and unexpectedly from epilepsy. Akaboshi et al. [12] functionally analyzed 27 variants and found that all markedly reduced enzyme activity; because residual protein level did not correlate with the wide phenotypic differences seen among families, they hypothesized additional modifiers drove disease severity.

To clarify what determines disease severity and the loss of enzyme activity, we characterized 12 SSADHD patients and measured the residual enzyme activity of 14 variants. Besides, we conducted extensive in silico studies on these variants, which can help clinical geneticists understand the predictive efficacy of in silico tools in assessing the degree of protein function disruption caused by the variants. The clinical data, in vitro data, and in silico data that we provided would be helpful for the interpretation of sequencing results by the ACMG/AMP guideline.

## Materials and methods

### Subjects

This study was approved by the ethics committee of Tianjin Children’s Hospital. Written consents were obtained from the subjects or their legal guardian. A total of 12 unrelated patients were recruited from Tianjin Children’s Hospital. Patients were diagnosed with SSADHD according to increased levels of GHB and the pathogenic variants in *ALDH5A1*, among whom patients 1 ~ 9 have been reported as clinical case series by us [13]. The clinical characteristics were collected during the follow-up.

### Whole exome sequencing

The DNA sequence of the *ALDH5A1* (NM_001080.3) was retrieved from the NCBI database.Whole exome sequencing was performed on DNA samples from the proband and/or parents. The Illumina was used for sequencing, with an average depth of 100-200x. We used the Hg19 human reference genome sequence, along with the Genome Analysis Tool Kit software, Annovar, Thousand Human Genome Database, dbSNP, and OMIM, to obtain and annotate the clinically relevant genomic variant data. To determine the variants’ locations, Sanger sequencing was performed on DNA samples from the proband and both of their parents. Chromas software was used to align the sequencing data using reference sequences from GenBank.

### Recombination and site-directed mutagenesis

We verified the predicted results of each software by constructing plasmids, transfecting, and performing multiple functional experiments. The wild-type (WT) *ALDH5A1* plasmid was cloned to pcDNA3.1 overexpression vector. Then, Fast Site-Directed Mutagenesis Kit (Tiangen) was used to generate mutant (Mut) *ALDH5A1* plasmids. For all constructs, the correct mutagenesis and plasmid integrity were confirmed by Sanger sequencing.

### Transient expression in Hela cell

The WT and Mut *ALDH5A1* were transiently transfected into Hela cells using Lipofectamine 2000 (Invitrogen). 48 h later, cells were harvested for western blot (Wb) and SSADH enzyme activity assay according to the manufacturer’s instructions. We set up three repetitive holes in every experiment and repeated the experiment at least three times.

### Wb analysis

Denatured proteins were separated by a 12% sodium dodecyl sulfate-polyacrylamide gel electrophoresis (SDS-PAGE) (Epizyme Biotech) and electro-transferred onto polyvinylidene fluoride (PVDF) membrane (Pall). After being blocked for 1 h with 5% evaporated milk and incubated overnight with relevant primary antibody (Abcam; diluted 1:20,000) in a 4℃ refrigerator and incubated 1 h with appropriate secondary antibody (Proteintech; diluted 1:5,000) at room temperature. The protein levels were normalized using the GAPDH (Zen-Bio; diluted 1:5,000). Membranes were scanned with Molecular Imager (BIO-RAD) and quantified by ImageJ software.

### SSADH enzyme activity assay

The enzyme activity of the WT and Mut proteins was evaluated using the fluorimetric assay described by Gibson and colleagues [14]. Total protein quantification of cell lysate was performed by using the BCA protein Assay Kit (Solarbio). An amount of 10 µl from the cell lysate was used for the enzyme activity assay; the incubations were performed for 90 min at 37 °C in 100 µl reaction volume containing 90 mmol/L Tris-HCl (pH 8.0, Solarbio), 0.2 mmol/L SSA (Toronto Research Chemicals), 3 mmol/L NAD^+^ (Solarbio) and 18 mmol/L DTT (Beyotime). Fluorescence was measured at an excitation wavelength of 355 nm and an emission wavelength of 470 nm with the Microplate Reader (BioTek). A NADH (Sigma) calibration curve ranging from 0 to 10 nmol/mL was performed for every experiment, and final results were quantified as nmol/mg/min. The experiment was repeated at least three times.

### In silico prediction and protein structure modeling

In silico tools (SIFT (https://sift.jcvi.org), PolyPhen-2 (http://genetics.bwh.harvard.edu/pph2), PROVEAN (http://provean.jcvi.org/index.php), and MutationTaster (http://www.mutationtaster.org)) were used to assess the putative pathogenicity of the detected variants in *ALDH5A1*. The PDB files and amino acid sequence of the WT SSADH monomer were obtained from the RCSB Protein Data Bank (http://www.rcsb.org). Among the protein models obtained by X-ray diffraction, we used 2W8O to predict the stability and structure changes of Mut proteins. We also used 2W8R and 2W8Q to predict protein structural changes and whether the variants potentially affected the binding function of SSADH with NAD^+^ or SSA [15]. We used the ProtParam tool (https://www.expasy.org/) to obtain various physical and chemical parameters of the Mut SSADH. The free energy of unfolding (ΔG) of variant monomers and tetramers was calculated by using the FoldX suite (version 5) for assessing stability [16]. The ΔΔG was the difference value between ΔG of Mut and WT proteins. As a basis for predicting changes in stability, ΔΔG > 0 means that the Mut protein verges toward destabilizing, and vice versa. The complete WT SSADH tetramer model was constructed using Alphafold2 [17]. The conformation with the highest fraction was selected, and various Mut tetramer models were constructed by the mutagenesis function of PyMol molecular graphics software. In addition, PyMol was also used to predict structural changes in Mut proteins. The hydrophilic index (HI) of amino acids was obtained from the study of Kyte et al. [18].

###  Variants classification according to ACMG/AMP [19]

According to ACMG/AMP guideline, the following codes were used for the assessment of pathogenicity classification: very strong (PVS1), strong (PS1, PS2, PS3, PS4), moderate (PM1, PM2, PM3, PM4, PM5, PM6), supporting (PP1, PP2, PP3, PP4), stand-alone (BA1), strong (BS1, BS2, BS3, BS4), and supporting (BP1, BP2, BP3, BP4, BP5, BP6, BP7) [20].

### Statistical analyses

The results of Wb and enzyme activity were analyzed using GraphPad Prism 6 software and presented as the mean with SEM using at least 3 independent experiments. A *t*-test was performed when comparing groups. A *P* value ≤ 0.05 was considered statistically significant. (* *P* < 0.05, ** *P* < 0.01, **** *P* < 0.001).

## Results

### Subjects information

Almost all the patients presented with psychomotor retardation, and half of them had seizures. As the gold-standard biomarker, elevated GHB was detected in all patients by urinary organic acids analysis. In 12 cases, 5 lost follow-up and 4 died. In total, only 3 (ID 1, 3, 9) were alive with long-term clinical data. At the last follow-up, patient 1 was 15 years old and had just 1 ~ 2 seizures per year. Besides, she suffered from severe hyperactivity disorder and struggled to speak well in social environment. Her motor ability was moderately impaired, but her intelligence was normal. Patient 3 demonstrated no abnormalities in epilepsy, psychiatric status, or motor function. Nevertheless, he was unable to manage daily living tasks, such as dressing or toileting. And his expressive language was restricted to single words. For patient 9, the prominent symptom was delayed language development. At age 3, she was assessed with moderate intellectual disability and had yet progressed beyond single-word utterances. Moreover, although seizures were infrequent, a follow-up electroencephalogram revealed abnormal epileptiform discharges. 13 *ALDH5A1* variants (1 splicing variant, 3 deletion variants and 9 missense variants) were detected in 12 probands (Supplementary Table 2). Clinical characteristics and variants were described in Table 1, Supplementary Tables 2 and 3. 8 patients carried the compound heterozygous variants, and the other 4 carried the homozygous variants.

**Table 1:** Clinical and genetic characteristics of different domains in this study

Domain	Variants (Amino acid substitutions)	Enzyme activity(%)	Patient	Phenotype	Prediction			
					SIFT	PolyPhen-2	PROVEAN	MutationTaster
Mitochondrial targeting sequence domain	c.85_116del (p.G29fs)	0.1	10	Intellectual disability, Motor developmental delay	-	-	-	Prediction Disease Causing
NAD+ bingding domain	c.398_399del (p.Q134*)	3.87	8	Seizure frequent, Intellectual disability, Motor developmental delay	-	-	-	Prediction Disease Causing
	c.515G > A (p.R172H)	29.7	-	Seizure, encephalatrophy	Damaging	Probably Damaging	Deleterious	Prediction Disease Causing
	c.638G > T (p.R213L)	7.4	8	Seizure frequent, Intellectual disability, Motor developmental delay	Damaging	Probably Damaging	Deleterious	Prediction Disease Causing
	c.691G > A (p.E231K)	41.4	1, 5, 10	1: Seizure: Infrequent, 10-year seizure freedom; Intellectual disability: Below elementary-school level; Communication: Need support in social communication; Severe hyperactivity disorder 5: Seizure Frequent, SUDEP in infancy; Intellectual disability; Motor developmental delay; Hypotonia, consciousness disorder 10: Intellectual disability, Motor developmental delay	Damaging	Benign	Deleterious	Prediction Disease Causing
	c.800T > G (p.V267G)	8.4	3	Intellectual disability: Little understanding of usual concepts and need support in daily living Communication: Deficient vocabulary and grammar	Damaging	Probably Damaging	Deleterious	Prediction Disease Causing
	c.865G > A (p.G289R)	8.3	7	Unexplained death; Intellectual disability; Motor developmental delay	Damaging	Probably Damaging	Deleterious	Prediction Disease Causing
	c.1529 C > T (p.S510F)	-0.2	2, 4, 5, 6, 9, 12	2: SUDEP in infancy; Seizure frequent; Intellectual disability; Motor developmental delay 4: Unexplained death; Intellectual disability; Communication disorder; Motor developmental delay; Hypotonia, hyporeflexia 5: SUDEP in infancy; Seizure frequent; Intellectual disability; Motor developmental delay; Hypotonia, consciousness disorder 6: Intellectual disability; Motor developmental delay; Hypotonia, hyporeflexia 9: Infrequent, < 1 seizure/year; Intellectual disability: Little understanding of usual concepts and need support in daily living; Communication: Need support in social communication 12:Intellectual disability; Motor developmental delay	Damaging	Probably Damaging	Deleterious	Prediction Disease Causing
Oligomerization domain	c.527G > A (p.G176E)	2.4	1	Infrequent, 10-year seizure freedom; Intellectual disability: Below elementary-school level; Communication: Need support in social communication; Severe hyperactivity disorder	Damaging	Probably Damaging	Deleterious	Prediction Disease Causing
	c.538 C > T (p.H180Y)	80.9	-	-	Tolerated	Benign	Neutral	Prediction Polymorphism
	c.545 C > T (p.P182L)	55.6	-	-	Tolerated	Probably Damaging	Deleterious	Prediction Polymorphism
Catalysis domain	c.983 C > A (p.A328D)	8.7	7	Unexplained death; Intellectual disability༛Motor developmental delay	Damaging	Probably Damaging	Deleterious	Prediction Disease Causing
	c.1105 C > G (p.R369G)	13.9	12	Intellectual disability; Motor developmental delay	Damaging	Probably Damaging	Deleterious	Prediction Disease Causing
	c.1274T > C (p.L425P)	2.8	11	Seizure; Intellectual disability༛Motor developmental delay	Damaging	Probably Damaging	Deleterious	Prediction Disease Causing

Abbreviations: “-”, not result

### The variants’ effects on protein structure and function

**Fig. 2:**
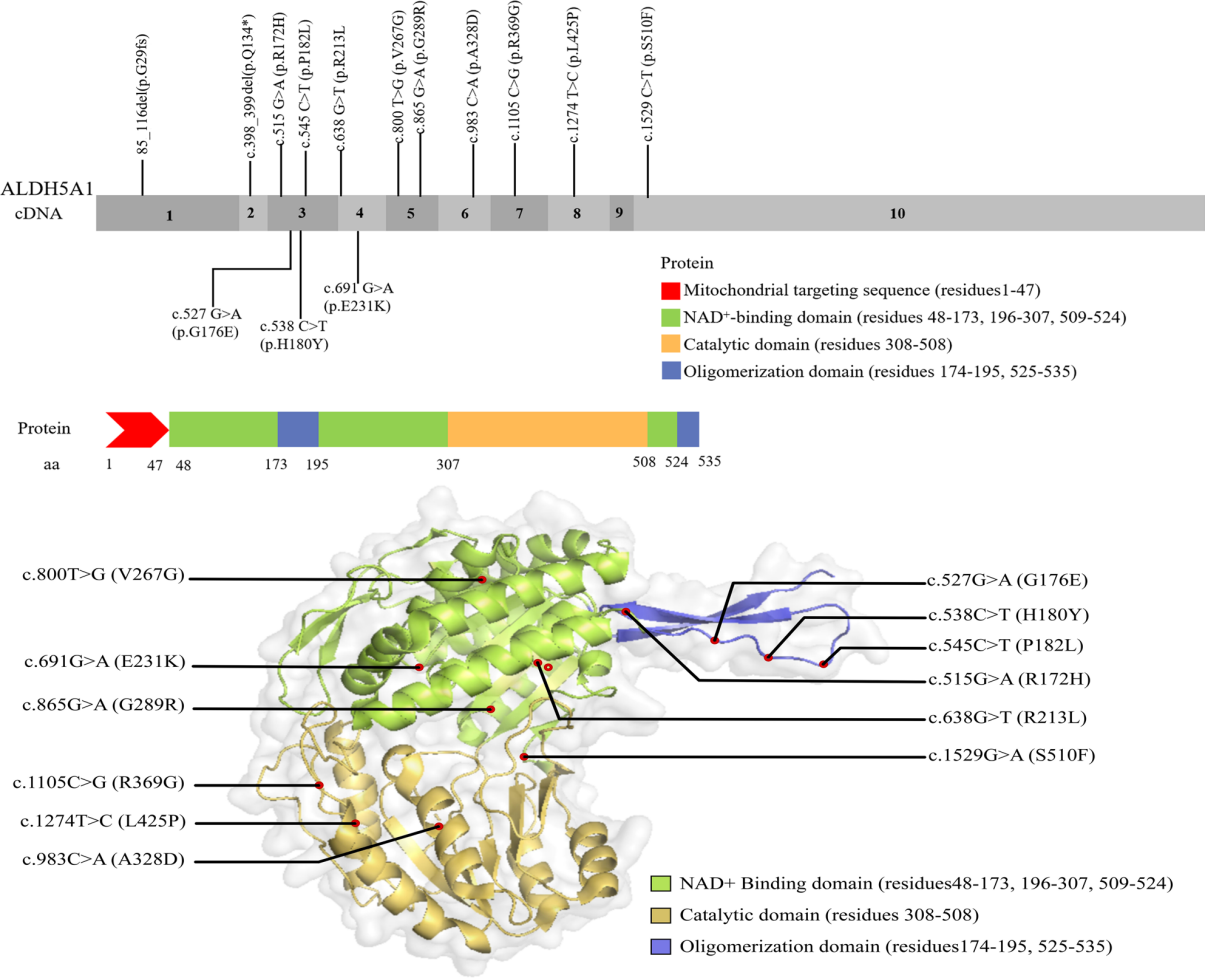
Schematic representation of the *ALDH5A1* and SSADH showing variants addressed in this study. Among all variants, the c.85_116del (p.G29fs) is located in the mitochondrial targeting sequence; the c.398_399del (p.Q134*), c.515G > A (p.R172H), c.638G > T (p.R213L), c.691G > A (p.E231K), c.800T > G (p.V267G), c.865G > A (p.G289R) and c.1529 C > T (p.S510F) are located in NAD+-binding domain; the c.527G > A (p.G176E), c.538 C > T (p.H180Y) and c.545 C > T (p.P182L) are located in oligomerization domain; the c.983 C > A (p.A328D), c.1105 C > G (p.R369G) and c.1274T > C (p.L425P) are located in catalytic domain

**Fig. 3:**
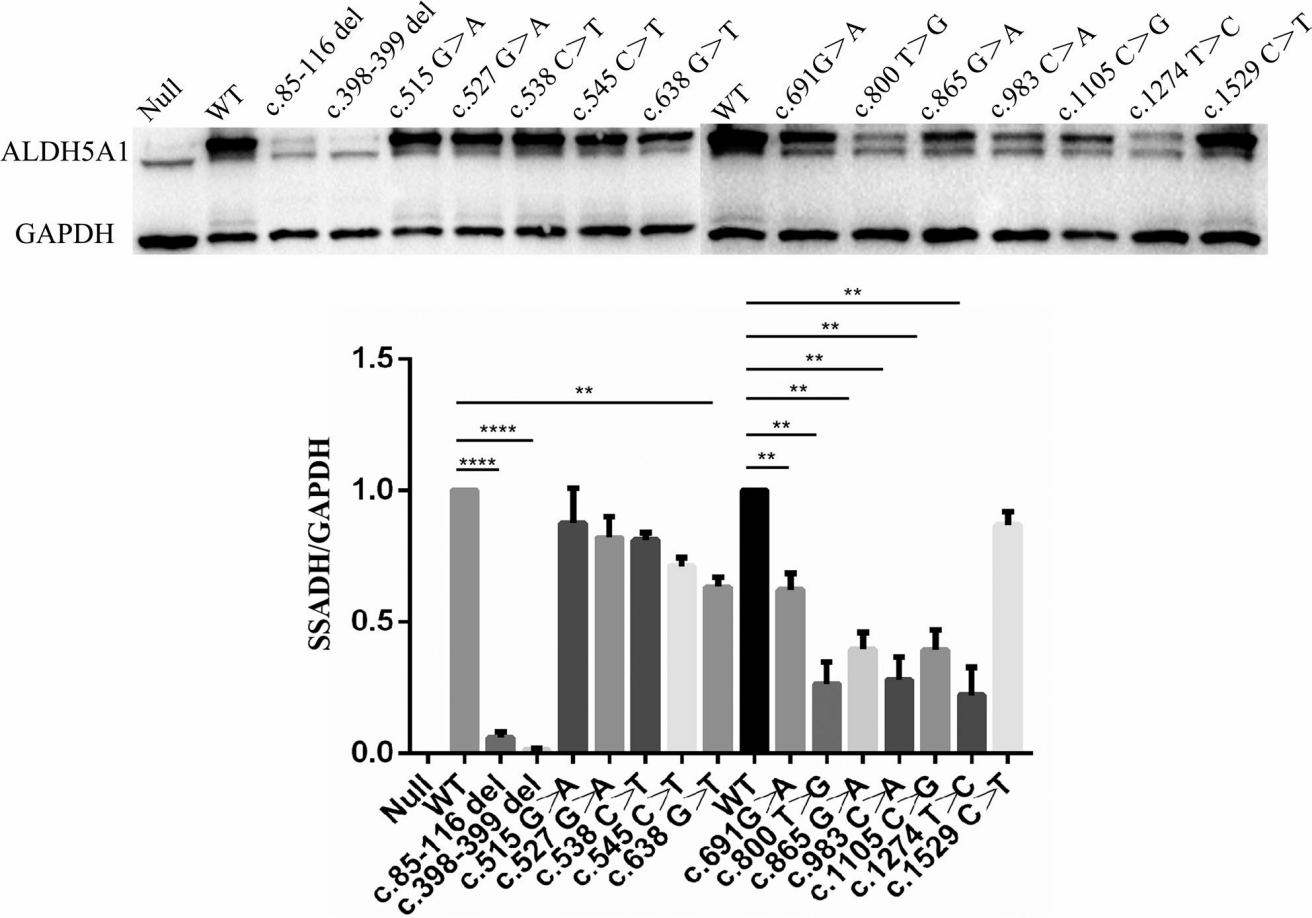
Wb analysis of WT and 14 Mut SSADH protein. Protein expressions in Hela cells overexpressing different cDNA constructs and data analysis of the protein expression levels. Presented as the mean with SEM using at least 3 independent experiments. P value of 0.05 or less was considered statistically significant (**P < 0.01, ****P < 0.0001). Null: non-transfected cell; the final results were standardized, (experimental group-null group) / WT group

**Fig. 4:**
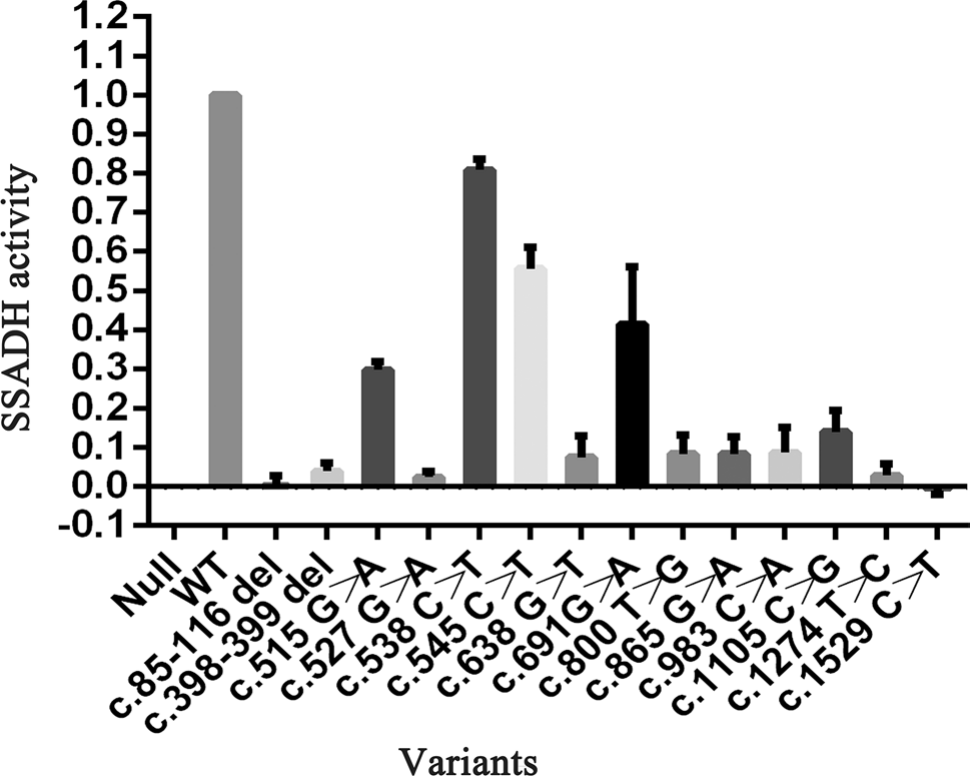
Enzyme activity of WT and 14 Mut SSADH protein in Hela cell

**Fig. 5:**
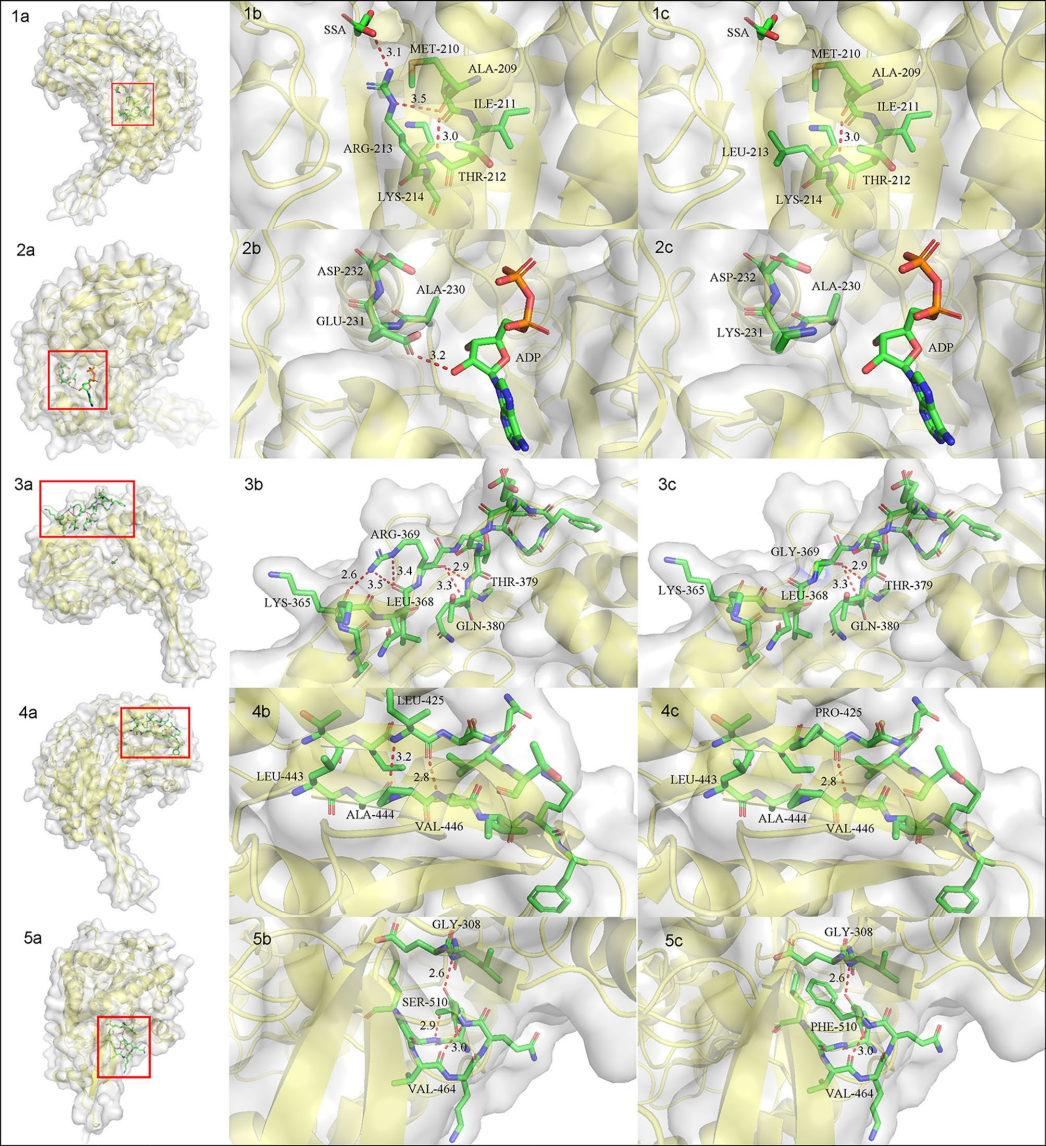
The red boxes in 1a-5a indicate the location of the variants relative to the proteins, and 1c-5c are the local conformation of the variants in the 3-dimensional models visualized by the variant proteins R213L, E231K, R369G, L425P, and S510F, respectively. 1b-5b are the references of the wild type in the same location as those variant proteins

All the 9 missense variants and 2 deletion variants (c.85_116del (p.G29fs) and c.398_399del (p.Q134*)) were included in the functional study. We also detected the homozygous variant c.515G > A (p.R172H) from an undiagnosed patient with epilepsy and encephalatrophy whose urinary GHB was normal; unfortunately, this patient succumbed to the condition; to clarify its pathogenicity and facilitate the carrier’s diagnostic work-up, we included this variant in the functional study. Finally, with the addition of two SNPs c.538 C > T (p.H180Y) and c.545 C > T (p.P182L) [7], they also were detected in patients 1 and 5, while not shown in the table, a total of 14 distinct variants in *ALDH5A1* were analyzed. These variants are distributed throughout *ALDH5A1* and span the entire SSADH protein (Fig. 2). The in silico predictions are summarized in Table 1. The Mut SSADH’s basic physical and chemical parameters (Supplementary Table 1), changes in HI and isoelectric points (pI) of Mut amino acids (Table 2), and quantitative assessment of stability (Table 2) are summarized. Mut protein expression data are shown in Fig. 3, and enzyme-activity data are presented in Fig. 4. The Mut proteins’ 3D structures are visualized in Fig. 5 and Supplementary figure.

**Table 2:** The change of hydrophilic index, isoelectric point and ΔΔG of each mutant SSADH proteins

Variants	Amino acid substitutions	HI-B	HI-A	pI-B	pI-A	ΔΔG	stability
c.515G > A	p.R172H	-4.5	-3.2	10.76	7.59	0.0676023	Neutral
c.527G > A	p.G176E	-0.4	-3.5	5.97	3.22	0.686939	Light destabilizing
c.538 C > T	p.H180Y	-3.2	-1.3	7.59	5.66	-0.22053	Neutral
c.545 C > T	p.P182L	-1.6	3.8	6.3	5.98	0.520347	Light destabilizing
c.638G > T	p.R213L	-4.5	3.8	10.76	5.98	-0.319228	Neutral
c.691G > A	p.E231K	-3.5	-3.9	3.22	9.74	-0.270613	Neutral
c.800T > G	p.V267G	4.2	-0.4	5.96	5.97	3.62253	High destabilizing
c.865G > A	p.G289R	-0.4	-4.5	5.97	10.76	8.06129	High destabilizing
c.983 C > A	p.A328D	1.8	-3.5	6	2.97	4.84899	High destabilizing
c.1105 C > G	p.R369G	-4.5	-0.4	10.76	5.97	2.88532	High destabilizing
c.1274T > C	p.L425P	3.8	-1.6	5.98	6.3	4.60824	High destabilizing
c.1529G > A	p.S510F	-0.8	2.8	5.68	5.48	7.02908	High destabilizing

Abbreviations: The HI-B and HI-A are the HI of amino acids before and after variant, respectively. The pI-B and pI-A are the pI of amino acids before and after variant, respectively. The ΔΔG is the difference value between ΔG of variant SSADH and wild type SSADH. The ΔΔG is the basis for accessing stability

#### Mitochondrial targeting sequencing domain

Of 14 variants in our study, only c.85_116del (p.G29fs) is located in this domain. The Mut protein’s physicochemical parameters differed markedly from those of the WT, and in silico prediction indicated that the truncation is damaging. Consistent with this, Wb analysis revealed a complete loss of full-length SSADH owing to the premature termination. Besides, c.85_116del (p.G29fs) abolished enzyme activity.

#### NAD^+^ binding domain

Among the variants in this study, the variants c.398_399del (p.Q134*), c.515G > A (p.R172H), c.638G > T (p.R213L), c.691G > A (p.E231K), 800T > G (p.V267G), c.865G > A (p.G289R) and c.1529 C > T (p.S510F) were located in NAD^+^ binding domain. Same with c.85_116del (p.G29fs), c.398_399del (p.Q134*) abolishes full-length protein and enzyme activity. Apart from c.691G > A (p.E231K), which three in silico programs classified as damaging, the remaining 5 variants were predicted pathogenic by all 4 tools.

The protein expression level (c.638G > T, 63.3%; c.800T > G, 26.2%; c.865G > A, 39.5%) and enzyme activity level (c.638G > T, 7.4%; c.800T > G, 8.4%; c.865G > A, 8.3%) of the 3 variants were significantly decreased. Protein expression level of c.515G > A (p.R172H) and c.1529 C > T (p.S510F) was essentially unchanged; however, p.R172H retained 29.7% of WT activity, whereas p.S510F nearly abolished it. Finally, c.691G > A (p.E231K) exhibited markedly reduced protein levels yet preserved 41.4% residual activity.

A better understanding of the impact of variants on SSADH might help us to gain a deeper comprehension. In the 3D models, Arg213 of SSADH was located near the “hole” that catalyzed SSA and formed hydrogen bonds with SSA, which was conducive to SSADH protein and SSA binding [21, 22]. The side chain of Leu was shorter than that of Arg and it could not form hydrogen bonds with SSA and Ala209 (Fig. 5 1a-1c). The drastic HI shift caused by c.638G > T (p.R213L) perhaps disrupts SSA recognition, abolishing substrate capture. The c.691G > A (p.E231K) produced the largest pI shift and reversed the polarity of the residue. Loss-of-function for c.691G > A (p.E231K) is partly explained by our 3D model: Glu231 normally donates a hydrogen bond to the ADP moiety of NAD^+^ [15], anchoring the cofactor within the NAD^+^ pocket; Lys231 abolishes this contact and destabilizes the complex (Fig. 5 2a–2c). Ser510 can form hydrogen bonds with Gly465, which disappeared in the Mut proteins 510Phe. The disappearance of hydrogen bonds may be an important reason for the function loss of c.1529 C > T (p.S510F) (Fig. 5 Fig. 5a and c). Consistent with this, significant ΔΔG also supports the highly destabilizing.

#### Oligomerization domain

The variants c.527G > A (p.G176E), c.538 C > T (p.H180Y), and c.545 C > T (p.P182L) map to the oligomerization domain, a three-β-sheet module that stabilizes the SSADH tetramer. Protein expression level of the 3 variants in the oligomerization domain was not significantly decreased. Enzyme activity for c.538 C > T (p.H180Y) and c.545 C > T (p.P182L) was not decreased significantly, whereas c.527G > A (p.G176E) abolished activity. In silico predictions for c.527G > A (p.G176E) were uniformly deleterious. In line with this trend, the c.538 C > T (p.H180Y) was predicted as benign by 4 tools; the c.545 C > T (p.P182L) was predicted as benign by 3 of 4 tools.

**Fig. 6:**
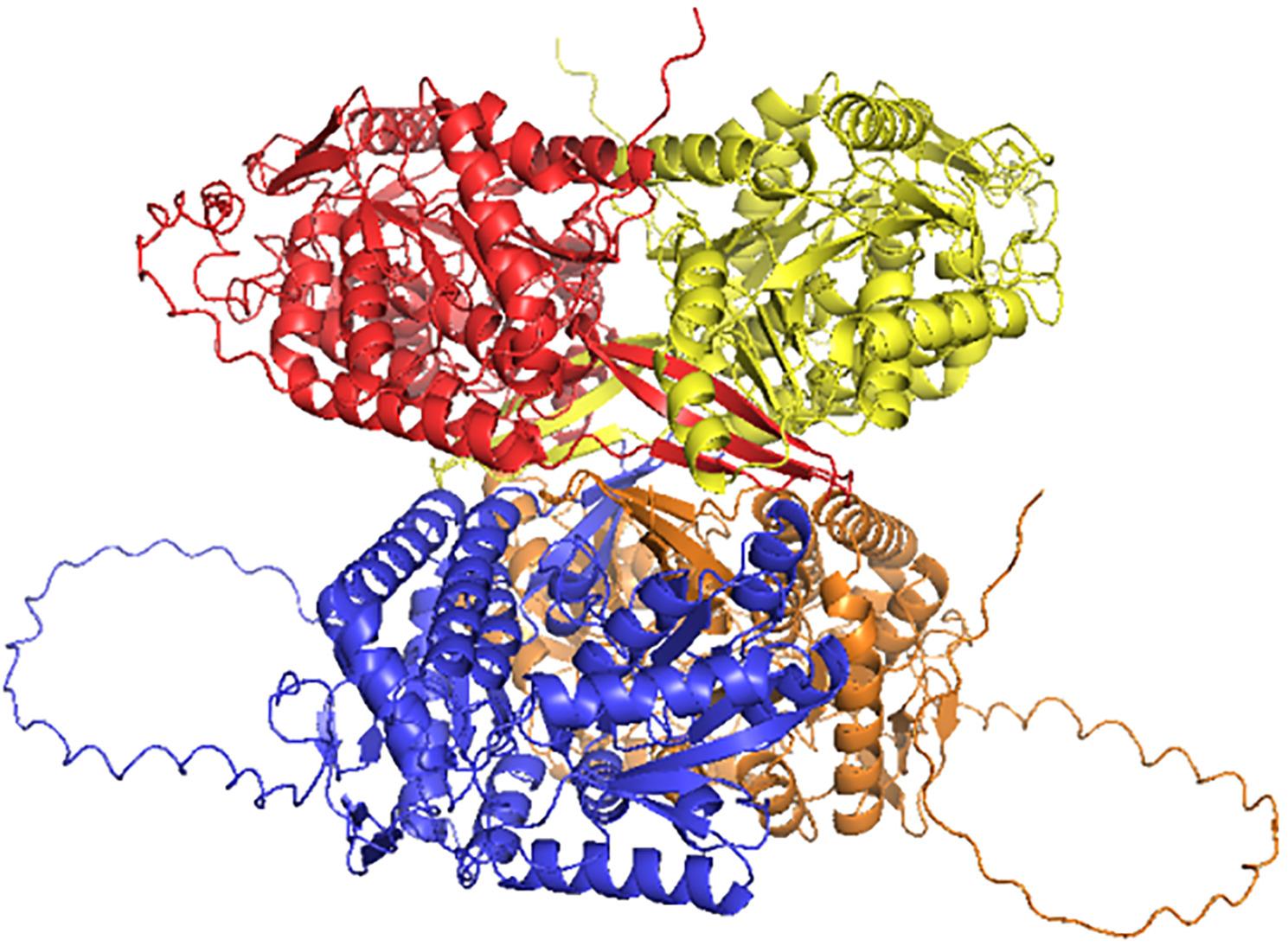
WT SSADH protein tetramers constructed by Alphafold2

To further clarify the influence of several Mut proteins in the oligomerization domain on the stability of SSADH tetramer, the WT SSADH tetramer model was constructed (Fig. 6). Following that, we constructed all the different isomorphic tetramers composed of the three Mut proteins mentioned above and the WT proteins. The ΔΔG of each variant tetramer was summarized in Table 3. According to the changes of ΔΔG, the damage of c.538 C > T (p.H180Y), c.545 C > T (p.P182L), and c.527G > A (p.G176E) on the stability of the tetramers increased in turn (Table 3). Moreover, with the increasing proportion of Mut monomers in the tetramer, the tetramer tended to be unstable (Table 3). The HI shift associated with c.545 C > T (p.P182L) was pronounced, whereas c.538 C > T (p.H180Y) replaced a basic residue with a neutral one, which may further perturb inter-subunit contacts.

**Table 3:** Tetramer stability of p.G176E, p.E231K, p.H180Y, p.P182L, and WT

Tetramers	ΔΔG(kcal/mol)	stability	Tetramers	ΔΔG(kcal/mol)	stability
EWWW	6.75	High destabilizing	YWWW	0.37	Neutral
EEWW	14.11	High destabilizing	YYWW	0.86	Light destabilizing
EWEW	14.58	High destabilizing	YWYW	0.48	Light destabilizing
EEEW	21.99	High destabilizing	YYYW	0.98	Destabilizing
EEEE	28.92	High destabilizing	YYYY	1.23	Destabilizing
EKKK	5.92	High destabilizing	LWWW	1.7	Destabilizing
EEKK	13.14	High destabilizing	LLWW	3.23	High destabilizing
EKEK	13.44	High destabilizing	LWLW	3.62	High destabilizing
EEEK	20.85	High destabilizing	LLLW	5.15	High destabilizing
KKKK	-0.62	Light stability	LLLL	6.79	High destabilizing

Abbreviations: E, K, Y, L, and W represent the monomers of G176E, E231K, H180Y, P182L, and WT, respectively. ΔΔG is the difference between the ΔG of the various tetramers constituted of the five monomers and the ΔG of the “WWWW”. The ΔΔG is the basis for accessing stability

#### Catalysis domain

The Mut protein c.983 C > A (p.A328D), c.1105 C > G (p.R369G), and c.1274T > C (p.L425P) were located in the catalysis domain. Protein expression level was significantly decreased (c.983 C > A, 27.9%; c.1105 C > G, 39.3%; c.1274T > C, 22.2%), and the enzyme activity level (c.983 C > A, 8.7%; c.1105 C > G, 13.9%; c.1274T > C, 2.8%) was also significantly decreased. All the in silico tools predicted marked instability for these variants. It is worth noting that both c.1105 C > G (p.R369G) and c.1274T > C (p.L425P) hydrogen bonds changed due to amino acid substitution, c.1105 C > G (p.R369G) had three fewer hydrogen bonds than the WT and the amino acid substitution of c.1274T > C (p.L425P) led to the loss of hydrogen bonds between the two β-sheets (Fig. 5 3a-3c, Fig. 4a and c).

### Pathogenic classification

Following ACMG/AMP guidelines, we accordingly assigned PVS1 (null variants), PM2/BA1/BS1 (allele frequency), PM3 (detected in trans), PP3/BP4 (in silico predictions), PP4 (phenotype specificity), and BP6 (supporting evidence of benign impact) to the 14 variants as appropriate (Table 4). Protein expression and residual enzyme activity served as the primary evidence for applying the PS3/BS3 codes. In accordance with ACMG/AMP guidelines, PS3 was assigned to 10 variants and BS3 to 1. PS3 code upgraded the evidence for 8 of the 14 variants; notably, c.800T > G (p.V267G), c.865G > A (p.G289R), c.983 C > A (p.A328D), c.1105 C > G (p.R369G), c.1274T > C (p.L425P) and c.1529 C > T (p.S510F) were reclassified from VUS to pathogenic (Table 4).

**Table 4:** Pathogenic classification according to ACMG/AMP guidelines

ID	Variant	Amino acid substitutions	ACMG/AMP codes							Classification
			PVS1 (Null variants)	PS3/BS3 (Function)	PM2/BA1/BS1 (Frequency)	PM3 (Trans)	PP3/BP4 (Prediction)	PP4 (Phenotype)	BP6 (Evidence)	
1	c.85_116del	p.G29fs	PVS1	PS3	PM2_Supporting	PM3	PP3	PP4	NA	Pathogenic
2	c.398_399del	p.Q134*	PVS1	PS3	PM2_Supporting	PM3	PP3	PP4	NA	Pathogenic
3	c.515G﹥A	p.R172H	NA	NA	PM2_Supporting	PM3	PP3	NA	NA	Uncertain significance
4	c.527G﹥A	p.G176E	NA	PS3	PM2_Supporting	PM3	PP3	PP4	NA	Pathogenic
5	c.538 C﹥T	p.H180Y	NA	BS3	BA1	NA	BP4	NA	BP6	Benign
6	c.545 C﹥T	p.P182L	NA	NA	BS1	NA	NA	NA	BP6	Likely Benign
7	c.638G﹥T	p.R213L	NA	PS3	PM2_Supporting	PM3	PP3	PP4	NA	Pathogenic
8	c.691G﹥A	p.E231K	NA	NA	PM2_Supporting	PM3	PP3	PP4	NA	Likely Pathogenic
9	c.800T﹥G	p.V267G	NA	PS3	PM2_Supporting	PM3	PP3	PP4	NA	Pathogenic
10	c.865G﹥A	p.G289R	NA	PS3	PM2_Supporting	PM3	PP3	PP4	NA	Pathogenic
11	c.983 C﹥A	p.A328D	NA	PS3	PM2_Supporting	PM3	PP3	PP4	NA	Pathogenic
12	c.1105 C﹥G	p.R369G	NA	PS3	PM2_Supporting	PM3	PP3	PP4	NA	Pathogenic
13	c.1274T﹥C	p.L425P	NA	PS3	PM2_Supporting	PM3	PP3	PP4	NA	Pathogenic
14	c.1529 C﹥T	p.S510F	NA	PS3	PM2_Supporting	PM3	PP3	PP4	NA	Pathogenic

Abbreviations: NA, not applicable

## Discussion

SSADHD is a rare neurometabolic disorder with onset in the late infantile period and early childhood, but may be diagnosed occasionally in adulthood [23]. We described 12 individuals with SSADHD in whom 14 distinct variants were identified (Supplementary Tables 2 and 3), including a novel pathogenic variant: c.1105 C > G (p.R369G). Our clinical data clearly demonstrated that the primary characteristics of SSADHD were intellectual disability, motor development delay, and seizures. Frustratingly, these non-specific neurological symptoms make the disorder easier to miss. *ALDH5A1* is the sole gene currently known to be associated with SSADHD [24]. Per expert consensus, SSADHD is diagnosed when either markedly elevated GHB or biallelic pathogenic *ALDH5A1* variants are present; ideally, both are demonstrated [25]. When developmental delays and intellectual disabilities remain unexplained, genetic testing should be the first-line diagnostic step [26]. In short, if clinical geneticists can confidently determine the pathogenicity of *ALDH5A1* variants identified by sequencing, further GHB metabolic testing can be omitted, sparing families and society substantial costs.

To establish the pathogenicity of *ALDH5A1* variants, generations of geneticists have invested considerable effort. For example, Pop et al. [8] studied the enzyme activity of several variants (c.527G > A (p.G176E), c.538 C > T (p.H180Y), c.545 C > T (p.P182L), c.691G > A (p.E231K), and c.1529 C > T (p.S510F)). To advance the genetic diagnosis of SSADHD, we have expanded the evidence base for variant pathogenicity. Yet even when pathogenicity data are available, knowing how to apply them correctly and judiciously remains an equally formidable challenge. The ACMG/AMP guidelines provide a practical template for variant classification, and under AMP’s Bayesian classification framework, these codes can now be quantitatively combined to estimate pathogenicity [19, 27, 28]. After integrating our protein expression and enzyme activity data with the additional evidence specified in the ACMG/AMP guidelines, we incorporated all criteria into the Bayesian Classification Framework to derive quantitative pathogenicity estimates. The results, presented in Section “Pathogenic classification”, represent a key contribution of this study.

Our research also has its limitations; our classifications still harbor zones of uncertainty. Firstly, we emphasize that our enzyme activity data reflect the catalytic capacity of the residual, expressed enzyme toward its substrate not the specific activity per enzyme unit. This means that the enzyme activity data carry greater weight than protein expression data. A direct comparison of c.691G > A (p.E231K) and c.515G > A (p.R172H) raises an apparent paradox: the former retains 41.4% activity, the latter only 29.7%. Yet c.691G > A (p.E231K) was found in patients 1, 5 and 10 (each unequivocally diagnosed by the gold standard elevation of GHB), whereas the child carried the homozygous variant c.515G > A (p.R172H) never exhibited increased GHB. This apparent contradiction is unsettling, yet viewing it through the lens of autosomal-recessive inheritance may point us toward the answer. Autosomal-recessive disease follows a single mechanism: biallelic loss-of-function that leaves residual activity below the threshold required for normal physiology [29]. Genes linked to autosomal-recessive disorders are typically haplo-sufficient. In patients 1, 5 and 10, the trans allele carrying c.691G > A (p.E231K) harbors 3 variants—c.527G > A (p.G176E), c.1529 C > T (p.S510F), and c.85_116del (p.G29fs)—that severely impair enzyme activity. In patient 1, the c.691G>A (p.E231K) is in cis with the c.545C>T (p.P182L), and c.538C>T (p.H180Y) is present both in cis and in trans with c.691G>A (p.E231K). In patient 5, two SNPs (c.538 C > T (p.H180Y) and c.545 C > T (p.P182L)) are present both in cis and in trans with c.691G > A (p.E231K). In short, the residual enzyme activity and even the clinical phenotype of patient are likely result from the combined effects of all the genetic variants. The residual activity conferred by the two c.515G > A (p.R172H) alleles may collectively exceed the haplo-insufficient threshold. It is admittedly crude that equating residual enzyme activity with allelic “dose”, while it offers a biologically plausible explanation: disease status is dictated by the combined residual activity of the multiple variants.

The 14 variants were distributed in all four domains, with about 50% in the NAD^+^ binding domain, 21.43% in the oligomerization domain, 21.43% in the catalysis domain and 7.14% in the mitochondrial targeting sequence domain. Moreover, most fatalities carried variants within the NAD^+^ binding domain, suggesting that lesions here confer greater severity. Brennenstuhl et al. [4] observed, pathogenicity rises toward the central and C-terminal regions of SSADH; the most deleterious alleles cluster in the NAD^+^ binding pocket, whereas variants residing in the catalytic domain display comparatively milder effects. These observations suggest that variants in certain domains may be correlated with a particular phenotype. During the study, we briefly entertained this possibility and tried to dissect such associations (Table 1), but the premise is clearly flawed. Once “moonlighting” effects are set aside, the combined residual “dose” contributed by the two alleles rather than their location per se, is the principal determinant of phenotype in this autosomal-recessive disorder [29]. We did not formally explore this quantitative “dose–response” relationship here, yet its existence remains entirely plausible.

The c.1529 C > T (p.S510F) may be a pointcut for the “dose-response” relationship. In our cohort, 6 of 12 patients harbor c.1529 C > T (p.S510F), 2 of 6 probands are homozygous varinat carriers, and 4 of 6 are compound heterozygous variants carriers. Moreover, c.1529 C > T abolishes enzyme activity in our assay, a finding that diverges somewhat from the ~ 5% residual activity previously reported [8, 30], we regard c.1529 C > T (p.S510F) as a null-activity variant. However, there were significant differences in the severity of phenotypes among these six patients; accordingly, the differing severity of these patients’ symptoms may be related to the damaging impact of the variants present in trans or other reasons, we will continue to conduct in-depth research in the future.

Although combining 3D visualization, stability predictions, and pI/HI shifts provided only weak insight into how individual variants cause loss of function, we nevertheless performed these analyses. Protein structure is characterized by a balance of hydrophobic and hydrophilic effects, and the structural stability is dependent largely on the hydrophobic action of amino acids [17]. In addition, due to differences in pI between amino acids, the substitution of amino acids also potentially changes the local charge of the protein molecules, and a change in charge may generate local instability [31]. Although the values may be overestimated, we still uncovered several interesting findings. Analysis of variants c.638G > T (p.R213L) and c.691G > A (p.E231K) underscores the critical roles of both SSA binding and the ADP motif for SSADH function. Then, how do variants (c.527G > A (p.G176E)) lying within the oligomerization domain cause disease when total protein levels are virtually unchanged? Can Variants in the oligomerization domain affect tetramerization? Based on the ΔΔG changes of the tetramer, c.527G > A (p.G176E) exerts the greatest impact on tetramerization, followed by c.545 C > T (p.P182L), while c.538 C > T (p.H180Y) has the smallest effect. Consistent with this trend, c.538 C > T (p.H180Y) and c.545 C > T (p.P182L) retained 80.9% and 55.6% activity, respectively, whereas c.527G > A (p.G176E) almost abolished enzyme activity. These cross-validating data support the reliability of our conclusions and offer some fresh perspectives for probing the underlying molecular mechanisms at the very least.

## Conclusion

In this study, we summarized the clinical and molecular phenotypes of 12 cases with SSADHD. Furthermore, we extended the follow-up data for 3 of the SSADHD patients. In 14 variants, one variant is original while the other 13 have been documented. We assessed the impact of 14 variants on protein expression and enzyme activity, and then used these data to assign ACMG PS3/BS3 codes as appropriate. After additional codes gathered under the ACMG/AMP framework, the pathogenicity classification of these variants was updated. Last but not least, we offered some fresh perspectives on illustrated molecular mechanisms. Our study also has limitations, as further extensive functional analyses are needed to clarify the pathogenicity and underlying molecular mechanisms of additional variants.

## Electronic supplementary material

Below is the link to the electronic supplementary material.

Supplementary Material 1

Supplementary Material 2

Supplementary Material 3

Supplementary Material 4

## Data Availability

The data supports the findings of this study are available within the article and its supplemental material or can be made available upon request.

## Abbreviations

ACMG/AMP: American College of Medical Genetics and Genomics/Association for Molecular Pathology
GABA: γ-gamma-aminobutyrate
GHB: γ-hydroxybutyrate
HI: Hydrophilic effects
Mut: Mutant
pI: Isoelectric points
SNP: Single nucleotide polymorphisms
SSA: Succinic semialdehyde
SSADH: Succinic semialdehyde dehydrogenase
SSADHD: Succinic semialdehyde dehydrogenase deficiency
WT: Wild-type
