# Supplementary material for: Succinic semialdehyde dehydrogenase deficiency: exploring the relationship between ALDH5A1 variants and molecular effect on SSADH
Source: Orphanet J Rare Dis. 2026 May 30;21:258. doi: 10.1186/s13023-026-04409-z (PMC13425955; doi:10.1186/s13023-026-04409-z)
Supplement: Supplementary file 1 — Supplementary Material 1 [file 13023_2026_4409_MOESM1_ESM.docx]

**Supplementary figure**

The 3-dimensional conformation of mutant proteins without definite conformational change.

All a indicate the location of the variants relative to the proteins, all b are the references of the wild type in the same location with those variant proteins, all c are the local conformation of the variants in the 3-dimensional models visualized by the variant proteins.

**R172H**


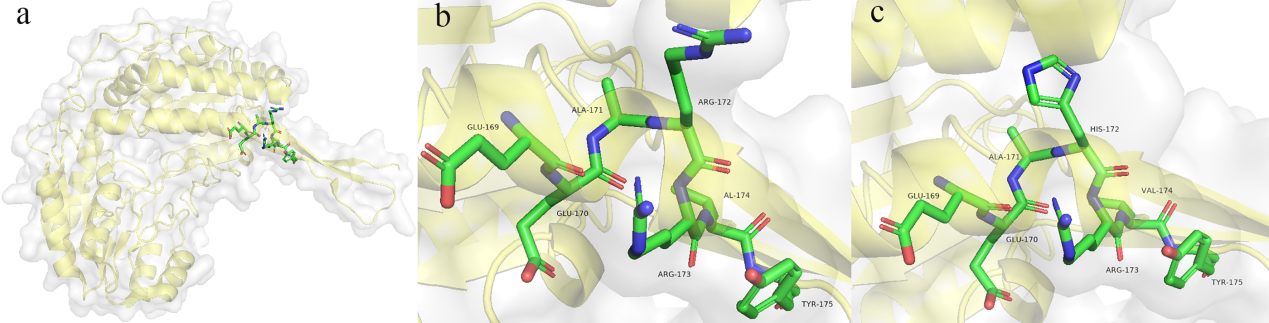


**G176E**

**
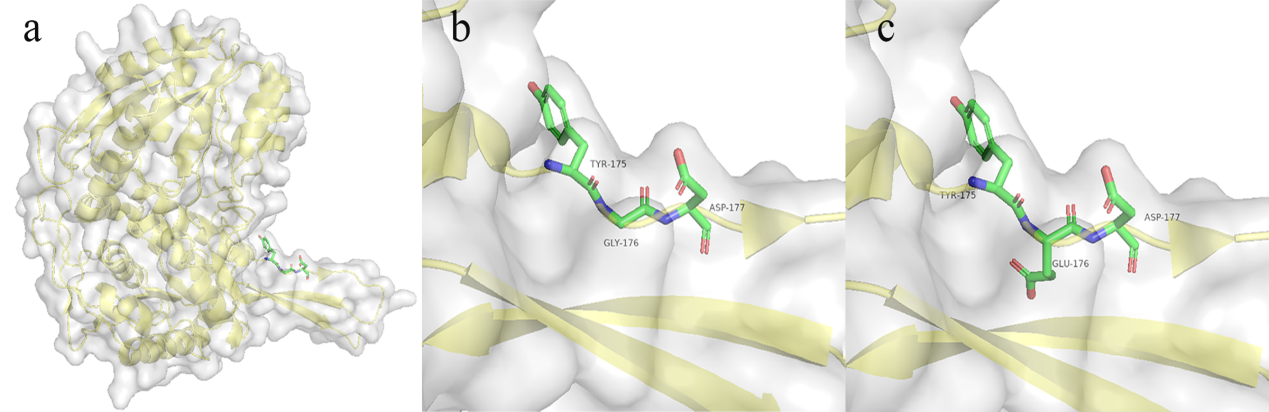
**

**
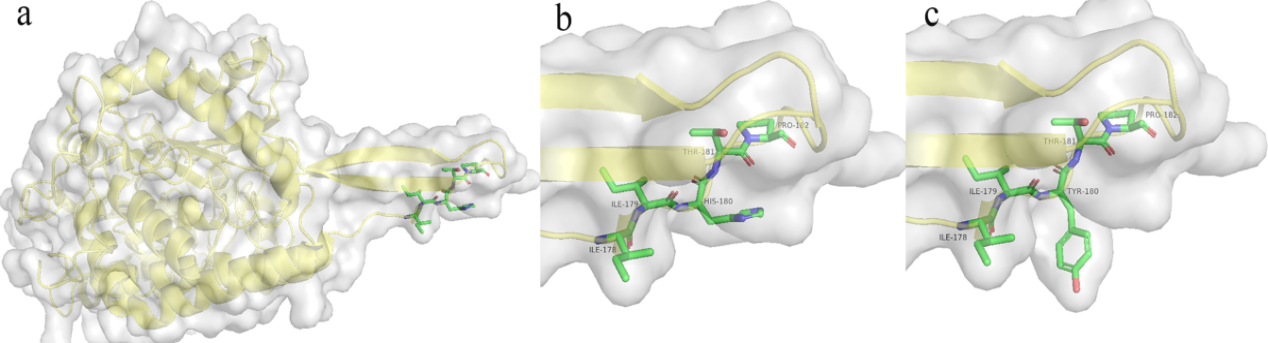
H180Y**

**
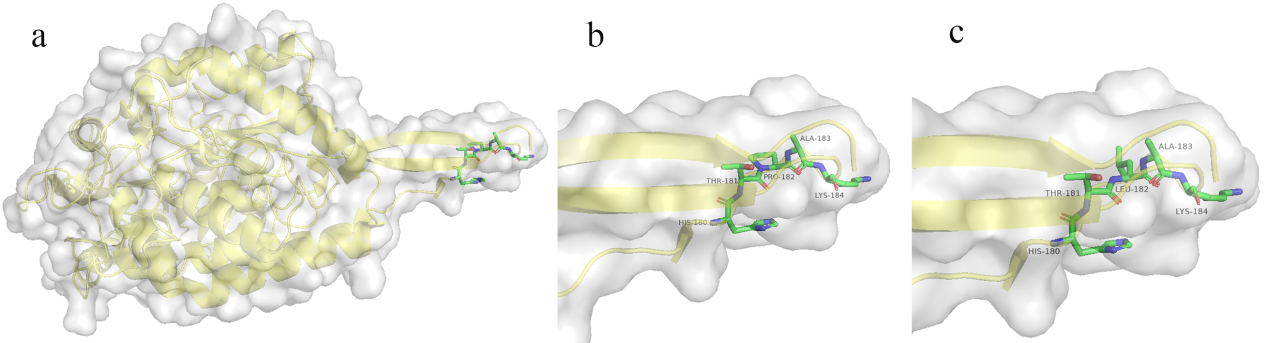
P182L**

**V267G**

**
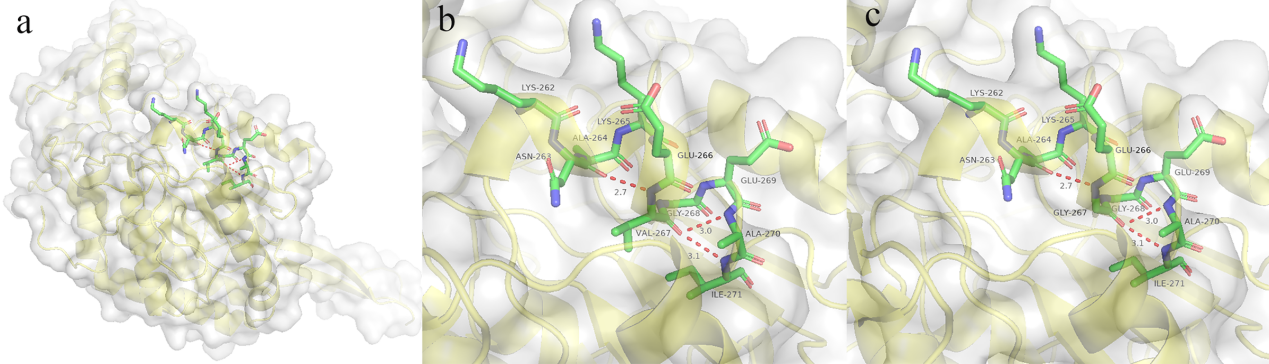
**

**G289R**

**
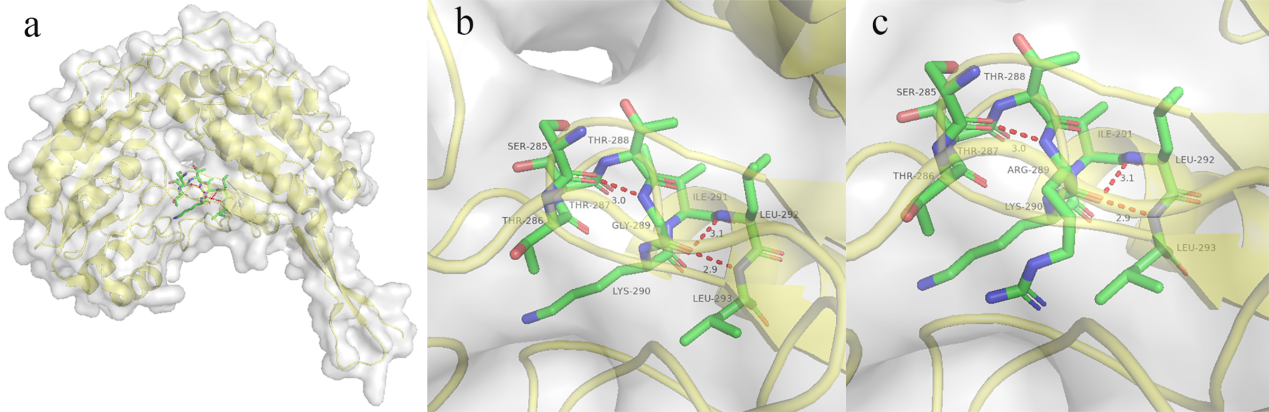
**

**A328D**

**
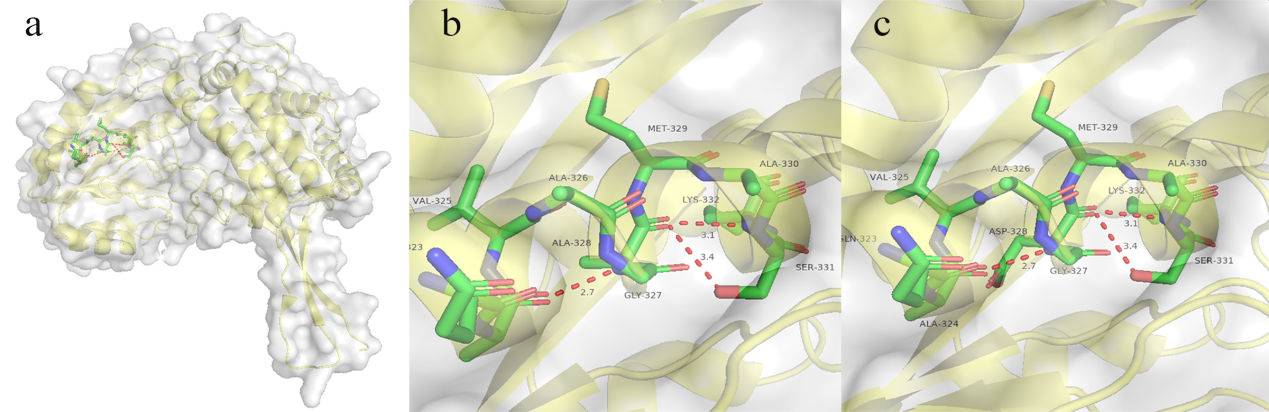
**
