# Supplementary material for: Succinic semialdehyde dehydrogenase deficiency: exploring the relationship between ALDH5A1 variants and molecular effect on SSADH
Source: Orphanet J Rare Dis. 2026 May 30;21:258. doi: 10.1186/s13023-026-04409-z (PMC13425955; doi:10.1186/s13023-026-04409-z)
Supplement: Supplementary file 2 — Supplementary Material 2 [file 13023_2026_4409_MOESM2_ESM.docx]

| Variants | Amino acid substitutions | Molecular weight | Theoretical pI | Instability index | Aliphatic index | Grand average of hydropathicity |
| --- | --- | --- | --- | --- | --- | --- |
| WT | - | 57214.76 | 8.62 | 39.01 | 86.5 | 0.01 |
| c.86-116del | p.G29fs | 13688.95 | 12.16 | 84.39 | 64.68 | -0.719 |
| c.398-399del | p.Q134* | 14253.6 | 10.74 | 65.04 | 79.63 | 0.045 |
| c.515G>A | p.R172H | 57195.71 | 8.52 | 37.79 | 86.5 | 0.013 |
| c.527G>A | p.G176E | 57286.82 | 8.52 | 39.39 | 86.5 | 0.005 |
| c.538C>T | p.H180Y | 57240.79 | 8.62 | 38.77 | 86.5 | 0.014 |
| c.545C>T | p.P182L | 57230.8 | 8.62 | 38.65 | 87.23 | 0.021 |
| c.638G>T | p.R213L | 57171.73 | 8.52 | 38.86 | 87.23 | 0.026 |
| c.691G>A | p.E231K | 57213.82 | 8.78 | 38.65 | 86.5 | 0.01 |
| c.800T>G | p.V267G | 57172.68 | 8.62 | 39.4 | 85.96 | 0.002 |
| c.865G>A | p.G289R | 57313.9 | 8.71 | 39.33 | 86.5 | 0.003 |
| c.983C>A | p.A328D | 57258.77 | 8.52 | 39.17 | 86.32 | 0.001 |
| c.1105C>G | p.R369G | 57115.62 | 8.52 | 38.65 | 86.5 | 0.018 |
| c.1274T>C | p.L425P | 57198.72 | 8.62 | 39.23 | 85.78 | 0 |
| c.1529G>A | p.S510F | 57274.86 | 8.62 | 38.05 | 86.5 | 0.017 |

Supplementary Table 1. Basic physical and chemical parameters of wild and mutant SSADH protein.
