# Supplementary material for: Succinic semialdehyde dehydrogenase deficiency: exploring the relationship between ALDH5A1 variants and molecular effect on SSADH
Source: Orphanet J Rare Dis. 2026 May 30;21:258. doi: 10.1186/s13023-026-04409-z (PMC13425955; doi:10.1186/s13023-026-04409-z)
Supplement: Supplementary file 3 — Supplementary Material 3 [file 13023_2026_4409_MOESM3_ESM.docx]

Supplementary Table 2. Clinical and genetic characteristics of included SSADHD patients.

| ID | Variants  (Amino acid substitutions) | Variant type | Zygosity | Sex | Age | | Seizure | Intellectual disability | Communication | Motor developmental delay | Other abnormalities |
| --- | --- | --- | --- | --- | --- | --- | --- | --- | --- | --- | --- |
|  |  |  |  |  | Onset | Follow-up |  |  |  |  |  |
| 1 | c.527G>A (p.G176E) | Missense | Compound heterozygous | F | 2y | 15y | Infrequent,  10-year seizure freedom | Below elementary-school level | Need support in social communication | No | Severe hyperactivity disorder |
|  | c.691G>A (p.E231K) | Missense |  |  |  |  |  |  |  |  |  |
| 2 | c.1529C>T (p.S510F) | Missense | Compound heterozygous | M | 12d | SUDEP in infancy | Frequent | Yes | NA | Yes | NA |
|  | chr:24403265-24566986del (p.?) | Deletion |  |  |  |  |  |  |  |  |  |
| 3 | c.800T>G (p.V267G) | Missense | Homozygous | M | 2y | 5y | No | Little understanding of usual concepts and need support in daily living | Deficient vocabulary and grammar | No | No |
| 4 | c.1344-2del (p.?) | Splicing | Compound heterozygous | F | 3m | Unexplained death | No | Yes | Yes | Yes | Hypotonia, hyporeflexia |
|  | c.1529C>T (p.S510F) | Missense |  |  |  |  |  |  |  |  |  |
| 5 | c.691G>A (p.E231K) | Missense | Compound heterozygous | M | 5m | SUDEP in infancy | Frequent | Yes | NA | Yes | Hypotonia, consciousness disorder |
|  | c.1529C>T (p.S510F) | Missense |  |  |  |  |  |  |  |  |  |
| 6 | c.1529C>T (p.S510F) | Missense | Homozygous | M | 3m | - | No | Yes | NA | Yes | Hypotonia, hyporeflexia |
| 7 | c.865G>A (p.G289R) | Missense | Compound heterozygous | M | 3m | Unexplained death | No | Yes | No | Yes | No |
|  | c.983C>A (p.A328D) | Missense |  |  |  |  |  |  |  |  |  |
| 8 | c.398_399del (p.Q134*) | Deletion | Compound heterozygous | F | 8m | - | Frequent | Yes | NA | Yes | NA |
|  | c.638G>T (p.R213L) | Missense |  |  |  |  |  |  |  |  |  |
| 9 | c.1529C>T (p.S510F) | Missense | Homozygous | F | 5m | 7y | Infrequent,  <1 seizure/year | Little understanding of usual concepts and need support in daily living | Need support in social communication | No | No |
| 10 | c.85_116del (p.G29fs) | Deletion | Compound heterozygous | F | 7m | - | No | Yes | NA | Yes | NA |
|  | c.691G>A (p.E231K) | Missense |  |  |  |  |  |  |  |  |  |
| 11 | c.1274T>C (p.L425P) | Missense | Homozygous | F | 9m | - | Yes | Yes | NA | Yes | NA |
| 12 | c.1105C>G (p.R369G) | Missense | Compound heterozygous | M | 7m | - | No | Yes | NA | Yes | NA |
|  | c.1529C>T (p.S510F) | Missense |  |  |  |  |  |  |  |  |  |

Abbreviations: d, day; F, female; m, month; M, male; NA, not available; “-”, loss of follow-up.
