# Supplementary material for: Succinic semialdehyde dehydrogenase deficiency: exploring the relationship between ALDH5A1 variants and molecular effect on SSADH
Source: Orphanet J Rare Dis. 2026 May 30;21:258. doi: 10.1186/s13023-026-04409-z (PMC13425955; doi:10.1186/s13023-026-04409-z)
Supplement: Supplementary file 4 — Supplementary Material 4 [file 13023_2026_4409_MOESM4_ESM.docx]

Supplementary Table 3. Basic information and bioinformatics software prediction of the *ALDH5A1* variants found in this study.

| Number | Variants | Domain | Exon | Amino acid substitutions | Prediction of damage | | | | Frequency |
| --- | --- | --- | --- | --- | --- | --- | --- | --- | --- |
|  |  |  |  |  | SIFT | PolyPhen-2 | PROVEAN | MutationTaster |  |
| 1 | c.85_116del | Mitochondrial targeting sequence domain | E1 | p.G29fs | -­ | -­ | -­ | Prediction Disease Causing | 1/26 |
| 2 | c.398_399del | NAD+ bingding domain | E2 | p.Q134* | -­ | -­ | -­ | Prediction Disease Causing | 1/26 |
| 3 | c.515G>A | NAD+ bingding domain | E3 | p.R172H | Damaging | Probably Damaging | Deleterious | Prediction Disease Causing | 2/26 |
| 4 | c.527G>A | Oligomerization domain | E3 | p.G176E | Damaging | Probably Damaging | Deleterious | Prediction Disease Causing | 1/26 |
| 5 | c.538C>T | Oligomerization domain | E3 | p.H180Y | Tolerated | Benign | Neutral | Prediction Polymorphism | SNP |
| 6 | c.545C>T | Oligomerization domain | E3 | p.P182L | Tolerated | Probably Damaging | Deleterious | Prediction Polymorphism | SNP |
| 7 | c.638G>T | NAD+ bingding domain | E4 | p.R213L | Damaging | Probably Damaging | Deleterious | Prediction Disease Causing | 1/26 |
| 8 | c.691G>A | NAD+ bingding domain | E4 | p.E231K | Damaging | Benign | Deleterious | Prediction Disease Causing | 3/26 |
| 9 | c.800T>G | NAD+ bingding domain | E5 | p.V267G | Damaging | Probably Damaging | Deleterious | Prediction Disease Causing | 2/26 |
| 10 | c.865G>A | NAD+ bingding domain | E5 | p.G289R | Damaging | Probably Damaging | Deleterious | Prediction Disease Causing | 1/26 |
| 11 | c.983C>A | Catalysis domain | E6 | p.A328D | Damaging | Probably Damaging | Deleterious | Prediction Disease Causing | 1/26 |
| 12 | c.1105C>G | Catalysis domain | E7 | p.R369G | Damaging | Probably Damaging | Deleterious | Prediction Disease Causing | 1/26 |
| 13 | c.1274T>C | Catalysis domain | E8 | p.L425P | Damaging | Probably Damaging | Deleterious | Prediction Disease Causing | 2/26 |
| 14 | c.1529C>T | NAD+ bingding domain | E10 | p.S510F | Damaging | Probably Damaging | Deleterious | Prediction Disease Causing | 8/26 |

Abbreviations: “-”, not result
